# Magnesium versus titanium headless compression screws for fixation of a Hoffa-type fracture - a biomechanical in-vitro study

**DOI:** 10.1186/s12891-025-08559-2

**Published:** 2025-04-02

**Authors:** Larissa Felten, Filippo Migliorini, Frank Hildebrand, Christian David Weber

**Affiliations:** 1 RWTH Aachen University, 52074 Aachen, Germany; 2 Department of Orthopaedics, Trauma and Reconstructive Surgery, University Hospital Aachen, Pauwelsstr. 30, 52074 Aachen, Germany

**Keywords:** Hoffa fracture, Biomechanics, Magnesium screws, Resorbable implant, Distal femur, Coronal fractures of the femur

## Abstract

**Background:**

Intraarticular (e.g., Hoffa-type) fractures are usually stabilized with titanium screws, which may necessitate later implant removal. The aim of this study was to compare the biomechanical strength and stability of magnesium and titanium screws.

**Methods:**

18 double-layer bone blocks were fixed with 18 one-layer bone blocks and divided into 2 groups based on the fixation method: magnesium screw fixation (Magnesium group, *n* = 9) and traditional titanium screw (Titanium group, *n* = 9). Compressive force was applied to the specimens orthogonally to the screw axis. First, axial stiffness was measured, and a cyclic loading test was performed, after 10, 100, 1000 and 10,000 cycles respectively, and the axial displacements were recorded. Finally, the specimens were loaded to failure.

**Results:**

There were significant differences between the groups with respect to axial stiffness and cyclic loading. The ultimate failure load was comparable. The average axial stiffness for the magnesium group was 326 ± 67 N/mm and for the titanium group 266 ± 72 N/mm (*p* = 0.031). The axial displacement relative to 100 N preload after 10,000 cycles in the magnesium group was 1.7319 ± 0.2261 mm and in the titanium group 2.6932 ± 0.5921 mm (*p* < 0.001). The average ultimate failure in the magnesium group was 920 ± 55 N and in the titanium group 944 ± 40 N (*p* = 0.293).

**Conclusions:**

Based on the results magnesium screws show at least a comparable strength and stability as titanium screws in this setting. This study provides support from a biomechanical perspective for the use of magnesium screws in Hoffa fractures.

**Clinical trial number:**

Not applicable.

## Introduction

The Hoffa fracture is a rare intra-articular fracture of the femoral condyle in the coronal plane, which is classified by the AO/OTA classification system as 33 B.3 and can be divided in three types with the help of the Letenneur classification [25, 30]. The most common Hoffa fracture type is the Letenneur Typ I fracture, which is a vertical fracture line parallel to the posterior cortex of the femur and involves the entire condyle [59]. That the fracture was first described by Hoffa in 1904, as it is reported in some papers, is a misbelief, because instead it was first described by Bush in 1869 [3, 49, 57]. The most common mode to cause this type of injury is a road traffic accident, especially motorcycle accidents, followed by a fall from height [18, 35, 51]. Usually, the lateral condyle is more often affected than the medial, but bicondylar fractures are found too [8, 11, 32, 35, 39]. Nonoperative treatment can lead to malunion, non-union, degenerative change and instability, which is why this fracture is usually operated today [26]. The aims of operative treatment are anatomic reduction of the articular surface, stable fixation and early mobilization [31]. The selection of the treatment approach and fixation material is based on individual preference of the surgeon. Usually, two 3.5 mm to 7 mm diameter headless compression, lag, partially threaded cancellous or cannulated cancellous screws in posteroanterior (PA) or anteroposterior (AP) screw fixation are used [12, 14, 40, 49, 51, 59]. Non-bioresorbable screws can cause several problems for example infection, pain, or foreign body sensation which sometimes require removal of the screws [41]. To the best of our knowledge, there are no studies that researched the use of magnesium screws in Hoffa fracture.

The aim of this study was to evaluate biomechanical behavior of magnesium to titanium screws in-vitro at initial state of implantation and transfer it to the clinical setting of Hoffa fractures with the help of literature. Hypothetically, it is assumed that titanium screws show superior performance in the indication specific in-vitro approach than comparable magnesium-based screws due to their higher strength.

## Materials and methods

### Preparing the test samples

**Fig. 1:**
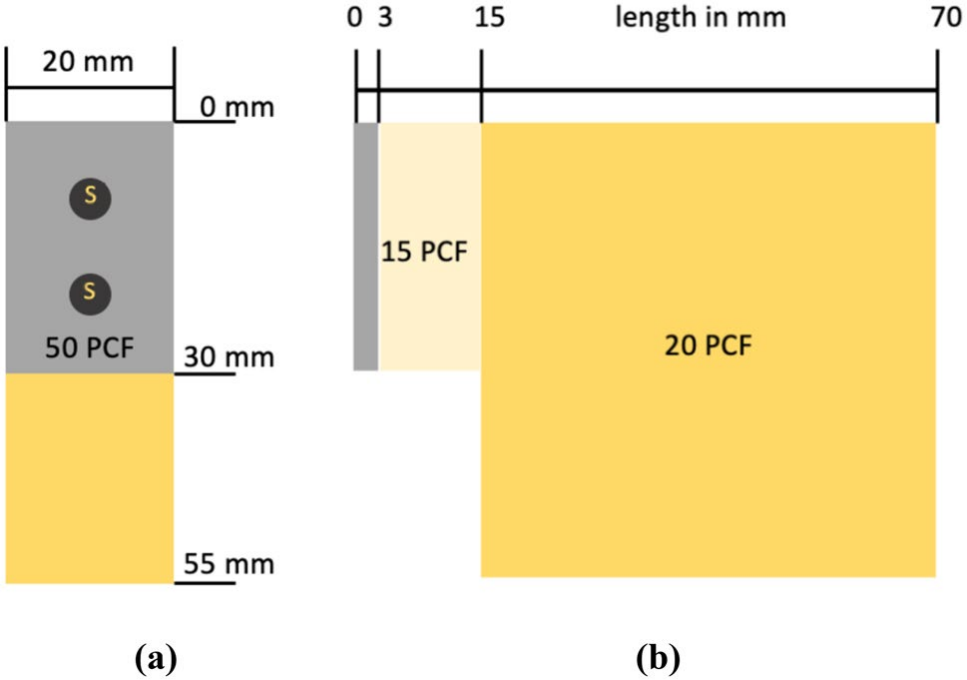
Schematic representation of the test samples; **(a)** the front view of the construct; **(b)** The lateral view. S = screw

**Fig. 2:**
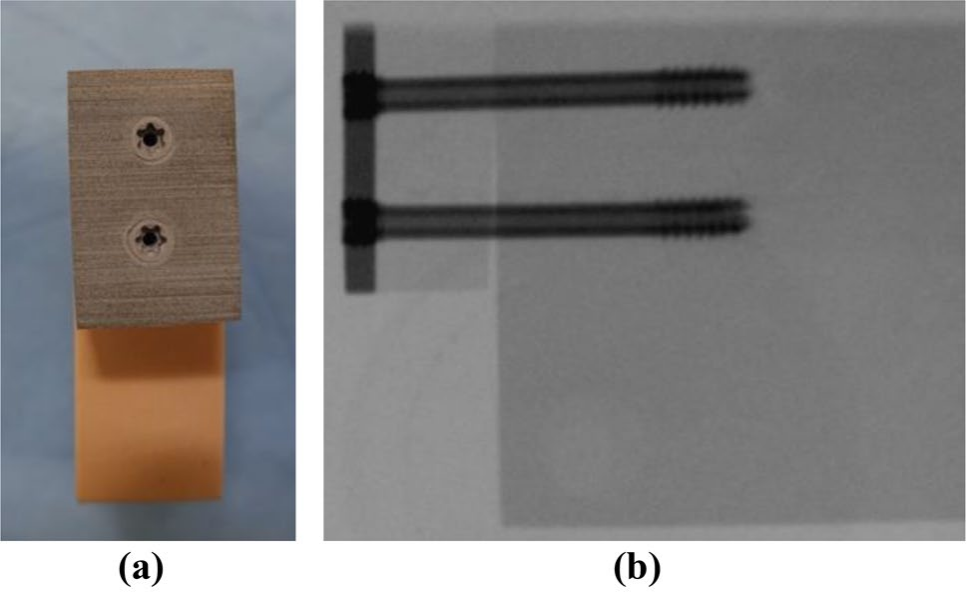
Prepared bone blocks with magnesium alloy screws before testing. **(a)** The frontal view; **(b)** X-Ray from the lateral view

**Fig. 3:**
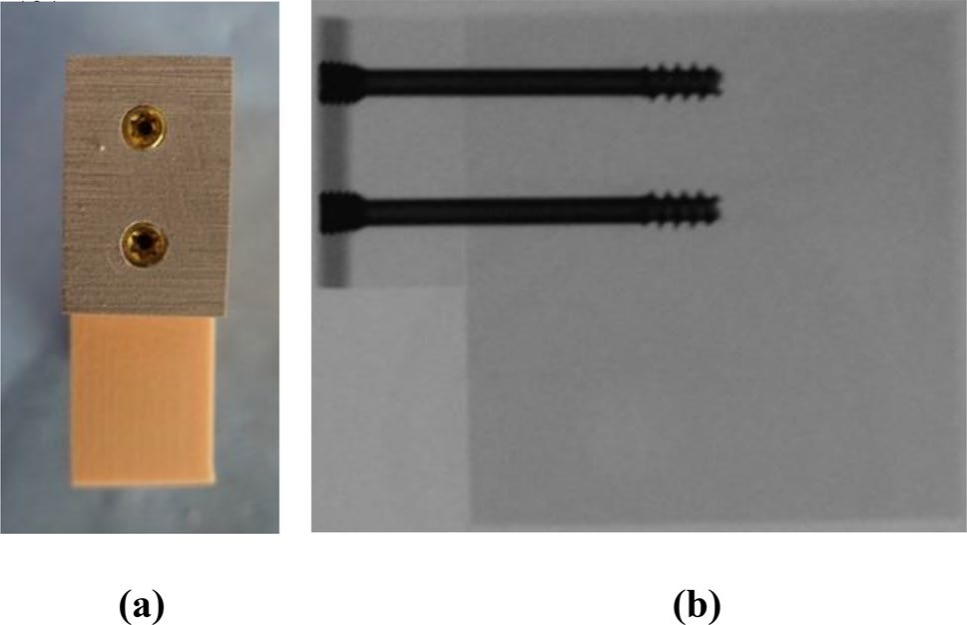
Prepared bone blocks with titanium screws before testing. **(a)** The frontal view; **(b)** X-Ray from the lateral view

**Fig. 4:**
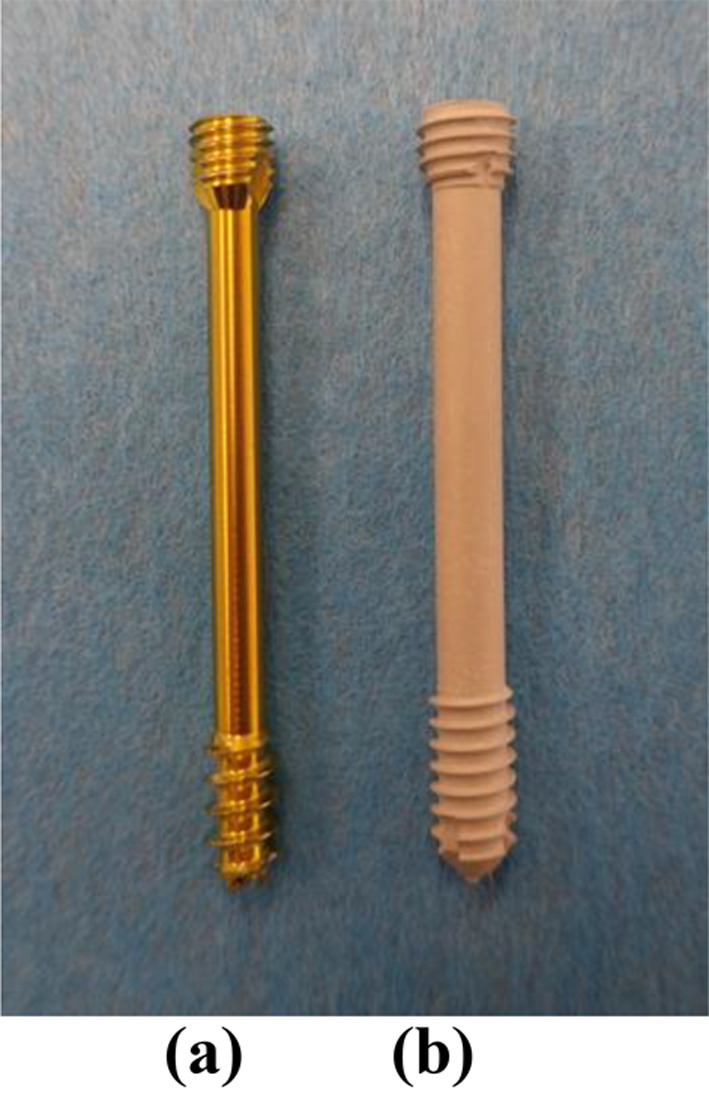
Test articles (compression screws). **(a)** 4.5 mm self-drill, headless compression screw made of titanium with the length of 44 mm, distal thread length of 9 mm and proximal thread length of 3.5 mm (Synthes GmbH, Oberdorf, Switzerland); **(b)** 5.0 mm magnesium-based CE certified mm.CS compression screw with the length of 44 mm, distal thread length of 11 mm and proximal thread length of 4.8 mm (Medical Magnesium GmbH, Aachen, Germany)

For the test samples, a bone block with a density of 20 Pounds per cubic foot (PCF) (SYNBONE^®^, Zizers, Switzerland) was sawn into eighteen blocks with the size of 55 mm X 55 mm X 20 mm. A two-layer bone block (50/15 PCF) (SYNBONE^®^, Zizers, Switzerland) was sawn into eighteen blocks with the size of 15 mm X 30 mm X 20 mm. The two-layer blocks were fixated with two screws on the 20 PCF blocks. The 20 PCF and 15 PCF densities were placed on top of each other, so that the 50 PCF layer can serve as the cortical bone (Fig. 1). For the fixation two parallel magnesium-based CE certified mm.CS compression screws Medical Magnesium GmbH, Aachen, Germany) screws were used. The screws are made of the WE43MEO magnesium alloy developed by Meotec GmbH (Aachen, Germany) and possess a PEO-surface modification. For this study, the selected screw variants are cannulated and have a diameter of 5.0 mm, a length of 44 mm, distal thread length of 11 mm and proximal thread length of 4.8 mm. The mm.CS compression screws were used in nine block pairs (Fig. 2) (Fig. 3). Two parallel 4.5 mm self-drill, headless compression screws made of titanium with the length of 44 mm, distal thread length of 9 mm and proximal thread length of 3.5 mm (Synthes GmbH, Oberdorf, Switzerland) were used in the other nine block pairs (Fig. 3) (Fig. 4). Care was taken to insert the screws at a distance of ≥ 10 mm to each other to prevent fracturing of the bone blocks [5]. To simulate a PA fixation method of a Hoffa fracture, the screws were inserted at the side of the smaller fragment (two-layer bone block). To create the right angle and make it easier to insert the screws, initially the smaller block was fixated to the larger bone block with two K-wires. It was important to make sure that the blocks almost laid flat on top of each other and were flushed with each other at the edges. The blocks were pre-drilled in the needed length. The K-wires were used as guides. Each of the two screws was screwed into the bone blocks over the K-wire, until the head was flush with the surface of the bone block. The K-wires were dragged out and a torque limiter was used to fully insert the screws, which was limited to 1.5 Nm.

### Biomechanical protocol

**Fig. 5:**
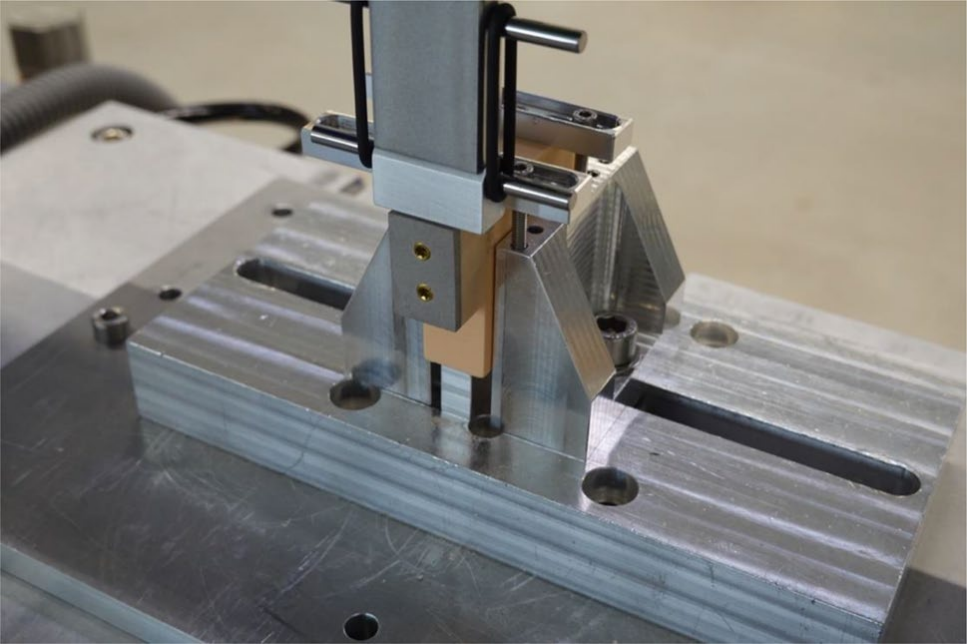
The test setup for biomechanical investigation

A class one materials-testing machine according to DIN EN ISO 376 (Dyna-Mess TP5, Stolberg, Germany) was utilized for the biomechanical tests and a compression strut was connected to a 5,000 N load cell, which recorded the applied axial compression force. The materials-testing machine is a class one force testing machine, according to DIN EN ISO 376. The relative resolution for the force is maximum 0.5% and for the displacement maximum 0.5%. The absolute resolution for the displacement is maximum 1.0 μm. The load was placed centrally on the double-layer bone block via stamp to evenly distribute the force application on the belonging fragment (Fig. 5). Initially, the stiffness test was performed. A compressive force was applied to the specimens orthogonally to the screw axis. The initial force was 75 N and the specimens were loaded up to 350 N. The stiffness of the specimens was subsequently calculated between 100 N and 300 N. The slope in the elastic region of the load displacement curve was recorded and calculated as the axial stiffness for the in-vitro test system. Subsequently, the cyclic two-point bending test, was performed. This bending test simulates the permanent load resulting from a common gait cycle. A 10,000-cycle, which are more cycles, than people walk on an average day, repeated loading test was applied to the specimen with a force ranging between 200 N and 600 N (valley/peak) at a frequency of 1 Hz, which is approximately the human walking speed [13, 47]. We think the vertical force represents the “worst-case”, because physiologically there would be a compressive force that woul help stabilizing the fragment during normal walking. The axial displacement was recorded, respectively after 10, 100, 1000 and 10,000 cycles. Finally, the load-to-failure test was performed. Destructive axial compression was loaded at a speed of 10 mm/min on each specimen. Because previous studies showed that a fracture step-off of 2 mm increases intra-articular pressure, the failure load was defined as a fracture displacement of 2 mm relative to the preload [36]. The loading protocol mainly followed two previous studies [19, 57].

### Statistical analysis

Statistical analysis was performed using the SPSS program (IBM SPSS Statistics software, version 29.0, Armonk, NY). Data were summarized as mean ± standard deviation (SD). For testing the data on normal distribution, the Shapiro-Wilk-Test was performed. If the data were normally distributed, the independent-samples t-test was performed and if not, the Mann-Whitney-U-test was performed. For testing the homoskedascity the Levene-Test was performed. If the homoskedascity was given, the independent-samples t-test was performed and if not, the Welch-test was performed. The significance level was set to *p* < 0.05.

## Results

In Table 1, the raw biomechanical data for the cyclic two bending test are listed.

### Axial stiffness test

**Fig. 6:**
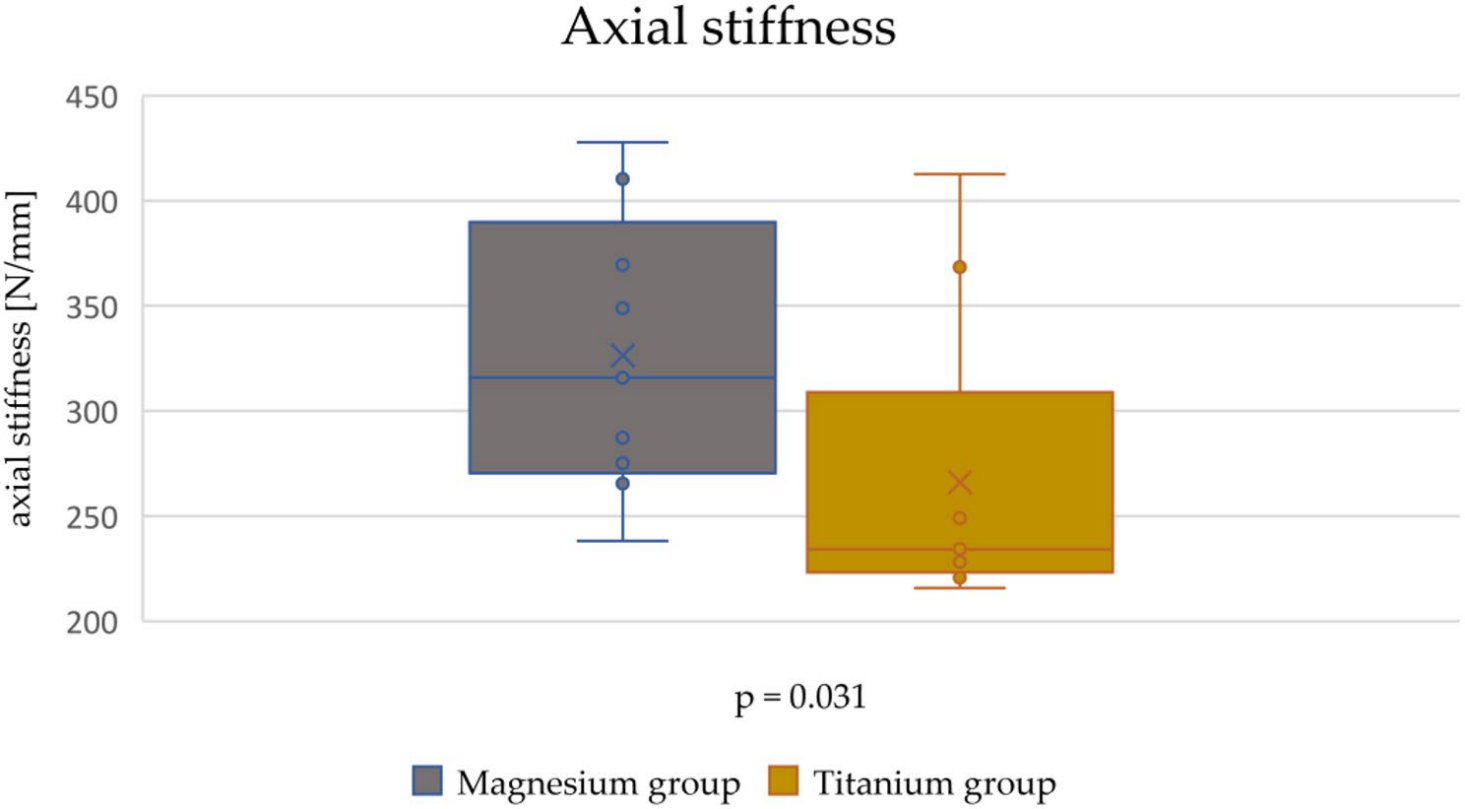
Box plot of the axial stiffness

The axial stiffness in the magnesium group was 326 ± 67 N/mm and in the titanium group 266 ± 72 N/mm. To calculate the p-value, the Mann-Whitney-U-Test was used, because the results of the titanium group were not normally distributed. The magnesium alloy group showed a significantly higher axial stiffness in this test than the titanium group (*p* = 0.031) (Fig. 6).

### Cyclic two-point bending test

The axial displacement in the magnesium screw group after 10, 100, 1000, 10,000 cycles were 0.6374 ± 0.0567 mm, 0.8356 ± 0.0819 mm, 1.1734 ± 0.1304 mm, and 1.7319 ± 0.2261 mm. The axial displacement in the titanium screw group after 10, 100, 1000, 10,000 cycles were 0.7197 ± 0.0645 mm, 1.0716 ± 0.1488 mm, 1.6828 ± 0.2917 mm, and 2.6932 ± 0.5921 mm. The magnesium group had significantly better results in the cyclic two point bending test after each cycle (as seen in Table 1). None of the specimens failed under the applied load. Increasing the load from 200 N to 600 N led to a displacement of 0.5685 ± 0.4142 mm in the magnesium screw group and 0.6001 ± 0.4004 mm in the titanium screw group. There was no significant difference between these groups (*p* = 0.119, significance level *p* < 0.05).

**Table 1:** Post Cyclic loading in the different groups

Group	Displacement [mm]				
	10 cycles	100 cycles	1,000 cycles	10,000 cycles	Delta 200 *N* to 600 *N*
Magnesium	0.6374 ± 0.0567	0.8356 ± 0.0819	1.1734 ± 0.1304	1.7319 ± 0.2261	0.5685 ± 0.4142
Titanium	0.7197 ± 0.0645	1.0716 ± 0.1488	1.6828 ± 0.2917	2.6932 ± 0.5921	0.6001 ± 0.4004
p-value	0.011	< 0.001	< 0.001	< 0.001	0.119

### Load-to-failure test

**Fig. 7:**
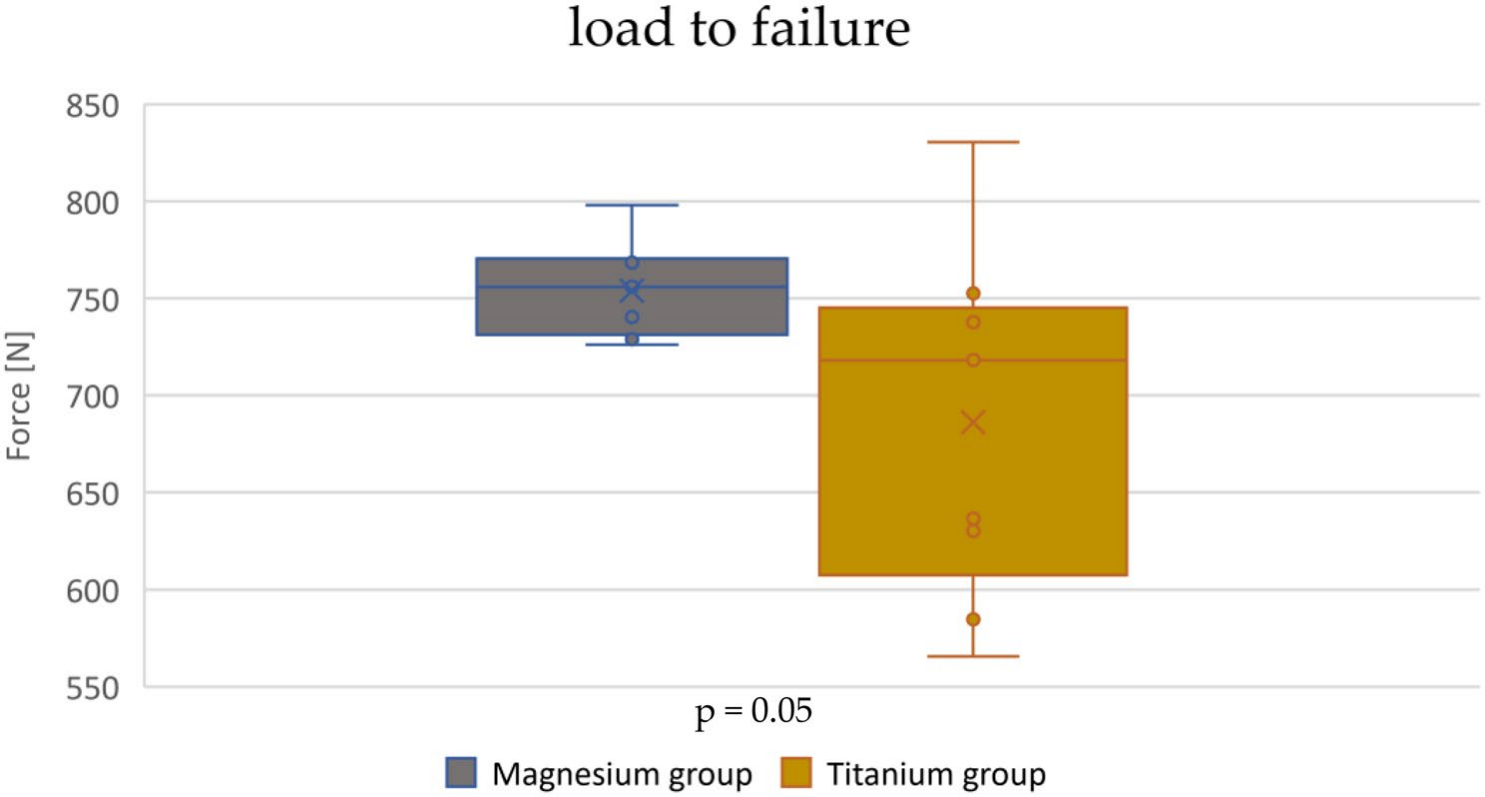
Box plot of the load-to-failure defined as displacement of 2 mm relative to the preload

The fracture displacement of 2 mm relative to the preload occurred in the magnesium screw group at a force of 754 ± 24 N. In the titanium screw group, the fracture displacement of 2 mm relative to the preload occurred at a force of 686 ± 87 N (Fig. 7). To calculate the p-value the Welch-Test was performed, because the variances were heterogeneous. There was no significant difference between these groups (*p* = 0.05, significance level *p* < 0.05).

## Discussion

In this study the biomechanical performance of magnesium-based and titanium screws in a Hoffa type in-vitro fracture model were compared. Based on the results, the magnesium screws showed superior biomechanical performance in-vitro, when compared to the titanium screws, except for the ultimate failure load. To the best of our knowledge, there are no studies that researched the use of magnesium screws in Hoffa fracture. Jarit et al. discussed two different fixation methods of 6.5 mm partially threaded cancellous screws in a Hoffa fracture in a similar biomechanical setting as conducted in this study [19]. One half of the screws were fixated in PA direction and the other half in AP direction and in each case two screws were used [19]. In the AP group, the displacement after 100 cycles was 1.13 ± 0.56 mm and after 10,000 cycles 1.36 ± 0.66 mm [19]. In the PA group, the displacement after 100 cycles was 0.57 ± 0.31 mm and after 10,000 cycles 0.67 ± 0.34 mm [19]. Screws fixated in the PA direction provide a significant higher stability than screws fixated in the AP direction [19]. It is easier for the surgeon to use the AP fixation method, but this should not influence the decision on using the PA fixation method, because the biomechanical advantages overweigh [1]. Both, the magnesium and titan group had a smaller displacement at 100 cycles, but a higher displacement at 10,000 cycles than the PA fixation group in the literature test from Jarit et al. The deviation maybe reasoned by the smaller diameter of the screws in our test [19]. Therefore, the findings of our study suggest that magnesium screws could provide a sufficient primary stability for fracture fixation of a Hoffa type fracture. For comparability, it should be considered that the diameter of the Magnesium screw is with 5.0 mm slightly larger than the titanium screw with a 4.5 mm diameter. Sahin et al. showed that magnesium screws can be as stable as titanium screws in a biomechanical setting even if the screws have the same design and size [46]. One indication for the used titanium screws with the diameter of 4.5 mm are distal femur fractures [50]. Since 3.5 to 7.0 mm diameter screws are used for Hoffa fractures, they also can be compared in a biomechanical setting to them with a larger diameter [12, 14].

Previous biomechanical studies discussed the ideal size and number of screws for fixation of Hoffa fractures [12, 14]. Hak et al. pointed out, that a two point fixation method prevent the rotation of the fracture fragment [14]. It was also shown that two 6.5 mm screws have significantly better stability than two 3.5 mm screws [14]. On the other hand, some advantages of screws with smaller diameters include usage in small fragments and the placement of more screws being placed in one fracture fragment [14]. Another advantage of smaller screws is less damage to the articular surface, if the screws have to be inserted in articular cartilage [1]. Large osteochondral lesions should be early detected and fixated [55]. Headless compression screws can reduce the damage to the articular surface compared to normal screws [6]. It was recommended to apply the screws at distance of ≥ 10 mm to prevent further comminution of the fracture fragment [5]. The selection of different screw types and fixation methods always subject to the surgeon’s personal treatment strategy, because of a high variability of fracture patterns, patients’ conditions and individual surgical preferences. Reith et al. surveyed patients who underwent hardware removal and analyzed common indications why it was performed, these include professional recommendation, local pain, impairment of function, foreign body sensation and the patients’ personal preference [41]. Infection or bacterial colonization of hardware is another indication for removal, especially late infections which are caused by constant hematogenous seeding of the implant from skin, respiratory, dental and urogenital infections [16]. Hardware removals may be associated with a variety of complications including infection, refracture, nerve injury or arthrofibrosis [7]. During search for hardware removal in Hoffa fractures in literature it was found out, that the hardware sometimes is removed when something else has to be operated in the knee, for example the anterior cruciate ligament (ACL) or removal of heterotopic ossification [17, 39]. Trikha et al. reported one case of hardware removal because of deep infection after four months [51]. Pires et al. reported one case of hardware removal because of soft tissue irritation [40]. In some studies, the indication for hardware removal was not further specified [4, 37]. Implant made from bioresorbable materials like magnesium alloys can avoid subsequent material removal surgery.

Post-traumatic osteoarthritis is one of the major complications after intraarticular fractures [4, 5, 20, 34]. Rhon et al. examined the risk of post-traumatic knee osteoarthritis after knee injury and found out, that around 10% of the patients developed early osteoarthritis [43]. Out of the people who developed osteoarthritis, 57% had at least one knee fracture [43]. Posttraumatic osteoarthritis is also a major indication for knee arthroplasty [45]. In a large prospective study from Denmark, Vestergaard et al. matched each patient with a knee fracture to five people without a knee fracture to examine the risk of total knee arthroplasty (TKA) after initial fracture treatment and throughout life [53]. They found out that patients with knee fractures have a 3.7 times greater risk of TKA within the first 3 years after the fracture, and the risk remains 1.6 times greater throughout their lifetimes [53]. After 20 years, 4% of patients with a distal femur fracture underwent TKA [53]. When a TKA is about to be performed, the screws which fixated the Hoffa fracture, can be removed prior to arthroplasty or at the time of the knee arthroplasty and sometimes left in place [27, 38]. There are several disadvantages of an implant removal at the time of knee arthroplasty [44]. Those include risk of wound necrosis in a patient with poor skin coverage or multiple prior scars, the increase of duration of the surgery with an increase need of anesthesia, an intraoperative fracture due to stress risers from implant removal and an increased risk of infection [44]. The cause of the infection can be an underestimated especially in low-grade-infections [15]. To prevent this, the implants can be removed in a prior operation and should be examined in a microbiological test [15]. The disadvantages of staged surgical approach before TKA are increased exposure to anesthesia, because of the second operation, delayed final surgery of TKA, infection, blood loss and costs [29, 52]. Especially if the screws were inserted years ago, implant removals may be more complex and associated with failure to removal the entire implant [29]. If the hardware is retained, the rate of surgical site complications, like infections or stiffness, may significantly higher [54]. Magnesium screws are bioresorbable, which means they do not have to be removed as they are degradable.

One concern for magnesium screws is the emission of hydrogen during degradation, which can lead to subcutaneous emphysema or initial impairment of bone healing [22, 24]. When the surface of the screws is modified, the degradation rate is low, avoiding any initial burst releases in gas formation [21, 42]. Accelerated degradation can lead to the risk of implant failure due to a lack of stability in Hoffa fractures [42]. With a PEO-surface modification, which is also used in our study, the degradation is slower [42]. In addition a patient with a Hoffa fracture is allowed to bear full weight with radiographic evidence of healing, which usually occurs by approximately 12 weeks of the postoperative period [4, 6, 9, 10, 28, 39, 49, 51].

Witte et al. compared four magnesium alloys and the associated bone response [56]. They noted that there was a significantly higher bone mass and a higher mineral apposition rate around degrading magnesium implants than around degrading polymer [56]. This can be explained by the Mg-ions released from Mg-based implants, which can promote osteostimulative processes [42, 56]. Kraus et al. compared two different magnesium pins and pointed out, that the slower degrading one enhanced bone neoformation around the implant [24]. As a conclusion they took this results to give evidence for good osteoconductivity and osteoinductivity of magnesium [24]. Magnesium screws can also exert beneficial effects on the formation of new blood vessels [58]. Another beneficial effect of magnesium screws is when they are fully degraded, they do not produce imaging artefacts during a CT or MRI scan like remaining metal implants do [2, 48].

The limitation of this study is typically defined by the in vitro nature in which bone substitute material is used instead of cadaveric femurs. On the other hand, cadaveric femurs often have osteoporosis, and a Hoffa fracture usually occurs in patients after a road traffic accident which are in the mean age younger than 40 years [11, 18, 20, 23, 33, 37, 51]. Since the comparative approach is used, the choice of sample material is only relevant to a limited extent. Comparability is achieved by testing both groups in the identical biomechanical setup. While the comparability of mechanical analysis results can potentially be improved by the standardized materials in relation to cadaver studies, this in vitro study with bone block materials is in any case limited to an initial state of fixation. The diameter of the magnesium screw was 5.0 mm and the diameter of the titanium screw was 4.5 mm. But since different diameter screws are used for Hoffa fractures, they also can be compared in a biomechanical setting to them with a larger diameter. The complexity of the loads acting in the body was also greatly simplified. Therefore, the absolute values are not fully transferable to clinical application, which is why the comparison to a gold standard, the titanium screw, was used.

## Conclusions

Based on this biomechanical in-vitro study on bone blocks, magnesium compression screws can provide at least comparable mechanical performance to titanium compression screws in a PA fixation method in Hoffa fractures. The current study provides support from a biomechanical perspective for the use of magnesium compression screws in Hoffa fractures.

## Data Availability

The datasets used and/or analysed during the current study are available from the corresponding author on reasonable request.
